# Tailoring Strength Training Prescriptions for People with Rheumatoid Arthritis: A Scoping Review

**DOI:** 10.1177/15598276221125415

**Published:** 2022-09-09

**Authors:** Michael L. Wu, Jasmin K. Ma, Karen Tsui, Alison M. Hoens, Linda C. Li

**Affiliations:** 8166 University of British Columbia, Vancouver, BC, Canada (MLW, AMH); 258853 Arthritis Research Canada, Vancouver, BC, Canada (JKM, LCL); and William Osler Health System, Brampton, ON, Canada (KT)

**Keywords:** resistance exercise, strength training, rheumatoid arthritis, prescription, guidelines

## Abstract

**Introduction**: Prescribing strength training (ST) for people with rheumatoid arthritis (RA) is complicated by factors (barriers and facilitators) that affect participation. It is unclear whether guidelines include recommendations beyond prescription parameters (frequency, intensity, time, type, volume, and progression) and adequately incorporate participation factors tailored to people with RA. **Objective**: To summarize available recommendations to aid in the tailoring of ST prescriptions for people with RA. **Methods**: Medline, Embase, and CINAHL databases and gray literature were searched for guidelines, recommendations, and review articles containing ST prescription recommendations for RA. Article screening and data extraction were performed in duplicate by two reviewers. **Results**: Twenty-seven articles met the inclusion criteria. The recommendations address RA-specific ST participation factors including: knowledge gaps (of equipment, ST benefits, disease), memory problems, the management of joint deformity, comorbidity, the fluctuating nature of the disease and symptoms (pain, stiffness, flares), fear avoidance, motivation, need for referral to other professionals, and provision of RA-specific resources. **Conclusion**: This review summarizes recommendations for tailoring ST prescriptions for people with RA. Future research is required to understand how pain, symptom assessment, and unaddressed ST participation factors like sleep and medication side effects can be addressed to support ST participation amongst people with RA.

“The physical, psychological, and social factors that affect strength training participation in individuals with RA makes prescribing strength training a complex and multifaceted endeavor.”

## Introduction

Rheumatoid arthritis (RA) is an autoimmune inflammatory disease affecting 1% of the Canadian adult population.^1,2^ Pathological immune processes lead to dysregulated inflammation of the synovial membrane, resulting in structural joint damage and joint pain.^1^ The systemic inflammation associated with RA often leads to other secondary complications including changes in body composition (e.g., rheumatoid cachexia with increased fatty infiltration into muscle), declines in functional ability, cognitive dysfunction, fatigue, as well as co-morbidities such as depression, cardiovascular disease, hypertension, dyslipidemia, and secondary osteoarthritis.^1,3^

Strength training is a safe intervention that can address many sequelae of RA. Strength training is done by sustaining or repeating muscular action with a goal to increase muscle strength and muscle mass.^4^ It can be done with the use of one’s body weight, elastic resistance bands, household items, machines, or free weights.^5^ In addition to increasing muscular strength, strength training has been shown to reduce pain,^6^ lower inflammatory markers,^7^ improve body composition (i.e., decreased fat mass, increased muscle mass),^8,9^ increase functional ability (e.g, walking performance),^6,7^ and lower cardiovascular and other comorbidity risk among individuals with RA.^10,11^ In addition to these benefits, even high intensity strength training has been found to be well tolerated by people with RA with no evidence of worsening disease activity nor radiological joint damage.^7,12^ In 2018, The European League Against Rheumatism (EULAR) published physical activity recommendations supporting strength training as an integral part of standard care in RA. Specifically, they provide strength training prescription recommendations (frequency, intensity, time, type, volume, progression; FITT-VP) and advise participation in strength training at least twice per week.^13^

Despite its broad benefits, only 1–14% of people with RA engage in regular strength training.^14,15^ Barriers to participation in strength training identified in people with RA include challenges with recall of exercise technique, knowing how to adapt the exercise routine during a flare, knowing how to distinguish RA-related joint pain from delayed onset muscle soreness, and fearing that exercise may trigger symptoms.^16^ Further, healthcare professionals including rheumatologists, nurses, and physical therapists acknowledge the importance of physical activity but the majority of those surveyed were uncertain on how to prescribe strength training to individuals with RA.^17,18^ While there is a focus on how to prescribe strength training based on FITT-VP parameters,^7,13,19,20^ it is equally important to highlight strategies that may facilitate the delivery of strength training prescriptions.^21,22^ The 2018 EULAR recommendations note that while the FITT-VP recommendations are well established, their feasibility amongst people with RA is not as well studied. Therefore, the purpose of this scoping review is to summarize current available recommendations from clinical practice guidelines, review articles, and recommendation papers that address participation factors to aid in the tailoring of strength training prescriptions for individuals with RA.

## Materials and Methods

The scoping review methodology was based on the Arksey and O’Malley (2005) framework^23^ which was updated by Levac et al.^24^ (2010). Reporting was guided by the Preferred Reporting Items for Systematic Reviews and Meta Analyses—Scoping Review (PRISMA-ScR) checklist.^25^ Scoping review methodologies are recommended for addressing exploratory research questions by “mapping” evidence to synthesize knowledge and determine gaps in the research area.^23,24,26^ Given the broad nature of the research question, a scoping review was conducted as opposed to a systematic review, which may be more suited towards answering a specific question addressing outcome measures of a certain treatment or practice.^27^ Furthermore, the Arksey and O’Malley (2005)^23^ scoping review framework includes an optional step in which stakeholders are consulted to offer their perspective on methodology and preliminary findings. We used an integrated knowledge translation approach,^28^ whereby patient and healthcare professional partners were engaged in the scoping review process. Specifically, seven patient partners with arthritis, recruited from Arthritis Research Canada’s Arthritis Patient Advisory Board,^1^ co-developed the research question, data extraction sheet, interpreted the data, and contributed to manuscript writing. The details of their involvement are described within each phase of the methods. The scoping review protocol was uploaded to Open Science Framework on July 10 2020 (https://osf.io/n4ygx/?view_only=8cbeb4acb6034606bb757e97ceb5f1c3at).

### Identifying the Research Question

This scoping review addressed the broad question, “what are the available recommendations for tailoring strength training prescriptions for people with RA?” Seven patient partners, two of whom are physical therapists, co-developed the research questions and identified specific sub-questions to guide the scope of the research question.^29^ The specific objective was to identify recommendations for tailoring strength training prescriptions for people with RA.

### Identifying Relevant Studies

A medical librarian was consulted to develop the search strategy. The search was completed in May 2020. MEDLINE (OVID), Embase, and CINAHL were searched using the following keywords: (1) terms for strength training programs included resistance training OR strength training OR weight lifting OR resistance exercise OR strength exercise (2); terms for population included rheumatoid arthritis (3); terms for study designs included recommendation OR clinical practice guideline OR CPG OR prescription* OR review. Please see Supplemental Material 1 for a sample search strategy.

In addition, we searched gray literature including Canadian Agency for Drugs and Technologies in Health (CADTH), PROSPERO, Physiotherapy Evidence Database (PEDro), Canadian Medical Association (CMA), Clinical Practice Guidelines Infobase, National Institute for Health and Care Excellence (NICE), OTseeker, Guidelines International Network, Turning Research Into Practice (TRIP), Physiotherapy Association of British Columbia (PABC), The Scottish Intercollegiate Guidelines Network (SIGN), and Google Scholar. Reference lists of key articles were also searched. Two content experts identified from the author list of key articles were consulted to confirm the final list of articles.

### Study Selection

Study inclusion criteria were (a) published articles and gray literature, (b) guidelines, recommendations, and review articles (with or without meta-analysis), (c) people with RA, (d) strength training prescription recommendations specific to RA. Exclusion criteria were articles (a) written in languages other than English, (b) that do not discuss a strategy for prescribing strength training or for addressing factors that affect strength training participation, (c) single studies. Patient and healthcare professional partners confirmed the criteria used for study selection were appropriate to answer the research question.

Following removal of duplicates, titles and abstracts were screened, and eligible articles were reviewed independently by JM and MW. Discrepancies were resolved by discussion and a third reviewer was involved (LL) if consensus was not reached.

### Charting the Data

Previously reported RA-specific participation factors from semi-structured interviews were used to develop the first draft of the data extraction sheet (Supplemental Material 2).^30^ A virtual meeting was held with the seven patient and healthcare professional partners to refine the data extraction sheet. Partners also suggested distinguishing recommendations based on individual characteristics such as age, disease severity, and strength training experience. An updated draft of the data extraction sheet was circulated by email to patient and healthcare professional partners for edits and approval. JM and MW independently and iteratively extracted information from the first seven articles and reached consensus on both the data extracted and the process of extraction. MW charted the remaining articles and JM checked for accuracy.

The following data were extracted from each article: year of publication, article title, type of article, number of studies analyzed (if a review), countries represented by authorship team (all represented countries were considered for articles with multi-country collaboration.), article objective, target audience, prescription parameters, recommendations that address strength training participation factors (knowledge, memory, stiffness, pain, flares, fatigue, joint deformities, co-morbidities, fluctuating nature of the disease, fear of causing further damage, motivation, access to resources, sleep, mental health, medication side effects, other), recommended adaptations based on individual characteristics (age, disease severity, strength training experience), and suggested future research directions. The Association of the Scientific Medical Societies Guidance Manual and Rules for Guideline Development was used to classify any identified guidelines as (a) a systematic literature review including a subsequent synthesis of the evidence and a structured consensus process completed by a representative committee (S3), (b) a systematic literature review and synthesis of the evidence only (S2e), (c) a structured consensus process completed by a representative committee only (S2k), or (d) an informal consensus process by a group of experts (S1).^31^ The full data extraction sheet can be found in Supplemental Material 3.

### Quality Appraisal

All guidelines (including expert recommendations, evidence-based guidelines, and consensus-based guidelines) were assessed using the Appraisal of Guidelines for Research and Evaluation Instrument (AGREE II).^32^ It contains 23 items encompassing 6 domains including scope and purpose, stakeholder involvement, rigor of development, clarity of presentation, applicability, and editorial independence.^33^ The quality of systematic reviews was evaluated using the 16-item AMSTAR 2 tool (A Measurement Tool to Assess systematic Reviews).^34^ Narrative reviews were not evaluated with AMSTAR 2. AMSTAR 2 has yielded evidence of acceptable inter-rater agreement for most items when tested on a sample of 54 systematic reviews across 3 pairs of raters.^34^ AGREE II and AMSTAR 2 scoring was done independently by JM and MW, and discrepancies were resolved through discussion. LL acted as a third reviewer to resolve discrepancies. While not appraised for quality, recommendations that were supported by sources other than guidelines or systematic reviews (e.g., non-systematic reviews) were flagged as limitations in the discussion.

### Summarizing and Reporting the Findings

Descriptive analysis was conducted on article characteristics. Key findings and recommendations were categorized into factors that affect strength training participation by the core research team (MW, JM, and LL). These recommendations and their categorizations were presented to patient and healthcare professional partners to ensure relevance to end-users.

## Results

### Characteristics of Included Studies

**Figure 1.**
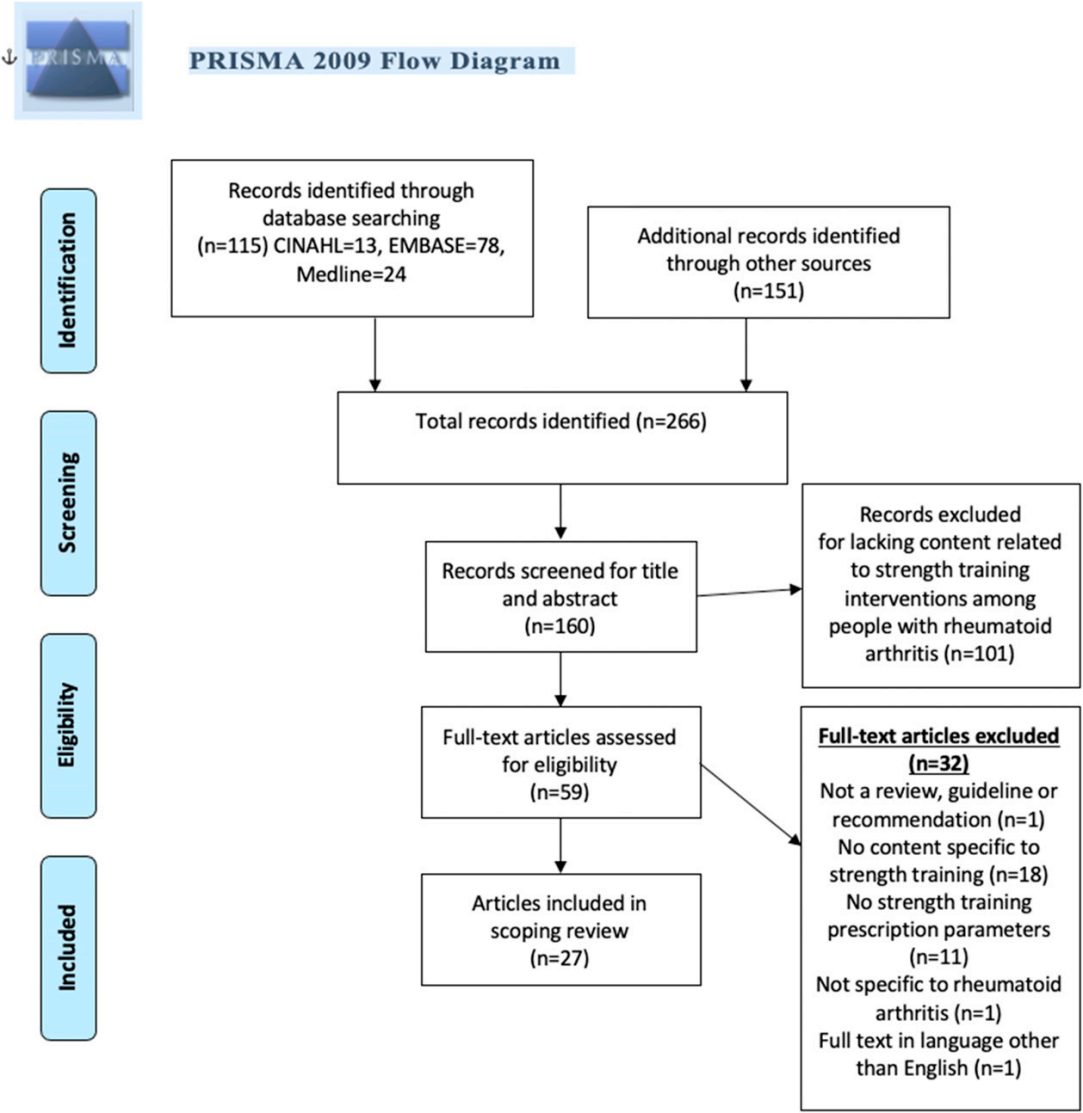
PRISMA Flow Diagram.

The search strategy retrieved 266 articles, 160 of which were screened for title and abstract. Of those, 101 were excluded with 59 articles proceeding to full text review. 32 full text articles were not eligible, leaving 27 articles eligible for review (Figure 1). Articles included in this scoping review were published between 1996 and 2019, with the majority (n = 15) published in 2010 or later (Table 1). Of the 27 articles, there were 12 narrative reviews, 5 systematic reviews, 5 consensus-based guidelines, 4 expert recommendations, and 1 evidence-based guideline. The majority of articles were targeted towards healthcare professionals (n = 20). Our full data extraction sheet is found in Supplemental Material 3.

**Table 1.** Description of Included Articles.

	Number	% of Studies
Year of publication		
1990–1999	1	4
2000–2009	10	37
2010–2019	15	56
Unspecified	1	4
Article types		
Systematic review	5	19
Narrative review	12	44
Evidence and consensus-based guideline (S3)	5	19
Evidence-based guideline (S2e)	1	4
Consensus-based guideline (S2k)	0	0
Recommendations by groups of experts (S1)	4	15
Countries^a^		
USA	8	30
Netherlands	5	19
Finland	3	11
France	3	11
Sweden	3	11
Switzerland	3	11
England	3	11
Denmark	2	7
Germany	2	7
Norway	2	7
Spain	2	7
Belgium	1	4
Canada	1	4
Cyprus	1	4
Ireland	1	4
Lebanon	1	4
New Zealand	1	4
Portugal	1	4
South Africa	1	4
Turkey	1	4
Wales	1	4
Target audience^a^		
Healthcare professionals	20	74
Patient organizations	1	4
Policy makers	1	4
Exercise professionals	2	7
Academics	1	4
Unclear	5	19
Discussed an RA-specific participation factor	17	63
Discussed population characteristics (e.g., age, RA severity, strength training experience)	17	63
Discussed pathophysiology of RA	21	78

^a^Some articles fulfilled multiple criteria for a given category. RA, rheumatoid arthritis.

### Recommendations for Tailoring Strength Training Prescriptions for People with RA

Several participation factors were addressed in the tailoring of strength training prescriptions for people with RA and are summarized in Table 2. These included knowledge gaps (of equipment, strength training benefits, disease), memory problems, the management of joint deformity, comorbidity, the fluctuating nature of the disease and symptoms (pain, stiffness, flares), fear avoidance, motivation, need for referral to other healthcare and exercise professionals, and provision of RA-specific resources. Specific prescription recommendations based on individual characteristics of age, RA disease activity, strength training experience, and performing strength training during a flare were also identified (Table 3). Tables 2 and 3 provide a summary of strength training prescription recommendations and content mapped to strength training participation factors and individual characteristics with their sources of evidence. The literature reviewed did not address participation factors including sleep, mental health, fatigue, and medication side effects. Synthesizing recommendations across the included literature, several recommendations were consistently supported (Table 2) and are summarized in Box 1.

**Table 2.** Recommendations for Tailoring Strength Training Prescriptions for People with Rheumatoid Arthritis.

Participation Factor	Recommendation or Content	Source(s)
Memory loss	Patients should be provided with clear and specific instructions (e.g., in a handbook or exercise plan), so they know exactly what the program entails	^35,36^
Pain	A long warm-up before exercise and a gradual cool down can help prevent pain	^58,37^
	Plan training around when pain medication is most effective	^37,13^
	Plan training for when pain is typically least severe	^13,38^
	If an exercise is painful, modify the movement (e.g., smaller range of motion), resistance, position, grip, repetitions, exercise or prescribe equipment that may be less stressful on the body (e.g., resistance bands vs machines)	^38,39^
	Adaptive devices can be used to reduce pain during exercise (e.g., padded gloves when holding weights, elevated blocks under the heels during squats, adapted shoes or insoles)	^39,40^
	If pain and fatigue are worse over the next few days, recommend doing less for the next session	^38^
	Monitor pain on a scale of 0–10	^41^
	Pain due to muscle soreness is usually harmless, but if it lasts more than 24 hours after exercise consider reducing the intensity	^13,40^
	Pain lasting more than 2 hours after exercise may be a sign of excessive exercise	^42,43^
	Pain location (i.e., in the muscle) can be used to distinguish normal delayed onset muscle soreness from potentially harmful joint pain	^39,44^
Stiffness	Plan the exercise when stiffness is lowest (e.g., avoid morning stiffness)	^38^
	Modify movements that are uncomfortable because of stiffness (e.g., decrease the range of motion required)	^39^
	Add activities that improve flexibility and reduce stiffness (e.g., stretching, range of motion, myofascial release such as foam rolling or consider recommending treatment with a physiotherapist)	^39,41,36^
Flares/increased disease activity	During a flare, focus on strengthening the muscles of least symptomatic joints	^45^
	During a flare, it’s important to encourage clients to stay active and moving; however, it is recommended to reduce the overall training intensity or volume	^37,38,58,35,45^
	During a flare, avoid high intensity exercise testing or assessment	^13^
	Strength training is discouraged during flare ups, although it is acknowledged that this is based on opinion and not linked to evidence	^5,44^
Joint deformities	Adaptive devices such as padded gloves, wrist orthoses, shoes and insoles can be used to lessen pain associated with joint deformities	^39,40^
Co-morbidities	Expect varying levels of fitness due to pulmonary and cardiovascular co-morbidities	^36,40^
	Avoid the Valsalva maneuver among individuals with high blood pressure	^37^
	Falls prevention exercises should be included for those with osteoporosis	^40^
	Screen for co-morbidities to determine if further medical clearance is warranted. The same relative and absolute contraindications for the general population apply to individuals with RA. RA may be associated with increased risk of cardiovascular, renal, pulmonary, immunological, and other types of health conditions that may affect suitability for exercise	^40,41,58,20,44,19^
	Clients may benefit from nutrition and exercise recommendations that target cardiovascular disease and obesity prevention	^41^
	Consider adequate calcium and Vitamin D intake for those at risk of osteoporosis	^41^
	Those with co-morbidities may require initial supervision	^35^
Fluctuating nature of the disease	Ask how the client is feeling (joint symptoms, pain, stiffness) before starting exercise to determine if the program needs to be adapted that day	^39,43,37,13^
	Activity can be separated into many short sessions during the day	^39,37^
	Limit strength training frequency to 2–3x/week and session time to less than 1 hour to support sustained participation	^44^
	Performing body weight movements during the warm-up can allow individuals to check how they are feeling before proceeding with the rest of the session	^39^
Fear of causing further damage	Progress slowly through the program while ensuring it is pain free (or as comfortable as possible) to help overcome fear of activity	^41^
Motivation	Choose activities that are enjoyable, convenient, provide variety, and become part of a normal routine	^37,39,43,20^
	Be active with a friend or join an exercise class	^38,20,35,19^
	Use behavior change techniques such as goal setting, action planning, and identifying benefits of strength training	^13,46,42,35,20^
	Stage of motivation: First stage focus on knowledge and motivation, second stage provide structure and supervision, third stage patients should be able to maintain their participation with adaptations as needed	^36^
RA-specific resources	The Arthritis Foundation was recommended for exercise examples and nutrition information	^39,38^
Referral to other healthcare professionals	Consider referral to an exercise professional with training or experience working with people with rheumatic disease	^37,38,47,39,46,48,36^
	Collaboration is needed between healthcare professional disciplines (e.g., physician, occupational therapy, dietician, cardiac rehabilitation) to ensure that patients are being supported and encouraged to pursue physical activity such as strength training	^39,5,19^
	Physical therapists and certified exercise professionals can teach exercise and breathing technique	^38,39^
Equipment knowledge	Free weights provide variety, better simulate activities of daily living, and develop muscle stabilization but may increase the risk of injury. Machines on the other hand control the movement in a single plane of motion	^44,39^
Knowledge of benefits	Increased muscle mass and decreased body fat	^47,38,44,19^
	Increased muscular strength	^39,42,49,13,46,5,40,41,48,58,20,44,35,19,50,51,52,43,53^
	Increased muscular endurance	^39,42,49^
	Increased functional ability through improvement in walking, greater ease rising from a seated position, or reduced risk of falls	^7,44,43^
	Psychological benefits such as improved mood, confidence, and reduced fear associated with movement	^39,48,50,51,52,49,53^
	Decreased inflammation or symptoms (e.g., pain, stiffness, and fatigue)	^39,47,38,46,5,40,41,58,7,54,50,51,49,53^
Knowledge of the disease	RA is a systemic autoimmune disease that is characterized by production of inflammatory agents that attack the synovium (membrane lining the joints) and often times other parts of the body	^40,37,47,38,39,5,41,48,42,58,45,17,50,19,36,52,43,55,53^
	Initially, RA typically affects smaller joints in the wrists, hands, knees, and feet and is often symmetrical	^39,17^
	It affects multiple structures and tissues including tendons, cartilage, bones, bursae of affected joints and can lead to deformities	^47,39,40,41,7^
	During a flare, the affected joints swell, and the joint capsule thickens	^37^
	RA affects different organs such as the cardiovascular, respiratory, central, and peripheral nervous systems	^47,39,41,48^
	Inflammation (often linked to the overproduction of inflammatory cytokines such as TNF-a) can lead to rheumatoid cachexia which is a condition characterized by a loss of muscle mass and concomitant increase in fat mass with a relative maintenance in weight	^39,44,19^
	Pain can be of a nociceptive character as a result of the inflammatory tissue process, but can also be neurogenic or widespread	^40,52,53^
	Joint instability is the result of elongated/lax tendons, ligaments and joint capsules	^40^
	Fatigue associated with RA is proposed to be related to pain, cerebral inflammation, and physical inactivity and/or lack of sleep	^40^

**Table 3.** Strength training prescription recommendations based on individual characteristics.

Characteristic	Recommendation	Source (s)
Age	For older adults, exercise on alternating days for adequate recovery and adaptation time	^44^
	For older adults, very light to light intensities can be used to improve strength among when beginning a training program (40–50% of the 1RM)	^13^
	For older adults, a single set of strength training exercise can be effective among older exercisers	^13^
Disease severity	In active disease or states of high inflammation, use medication to control inflammation before starting a training program	^38,19^
	Lower training intensity may be considered depending on radiographic joint damage and physical impairments	^36^
	In those with little inflammation but a lot of structural damage, focus initially on non-damaged joints. Load on damaged joints should happen progressively from ROM exercises to low load exercise	^19^
Training experience	Lower intensities can be used for those who are new to strength training	^39,13^
	If an individual has been inactive for a long time, they should check with their healthcare professionals to see if they have other health conditions	^38^
	Supervision and guidance can be helpful for those who are deconditioned or have little experience with training	^39,48^
	It is important for untrained individuals to exercise on alternating days for adequate recovery and adaptation time	^44^
	Complicated programs are not necessary for untrained individuals as they will benefit from most programs	^44^
	For untrained individuals, strength training frequently (e.g., daily) does not have enough benefits to justify the time commitment and reduced recovery time	^44^
	Start with body weight movements before adding weight to learn proper technique and body awareness	^39^

Abbreviations: RA, rheumatoid arthritis; RM, repetition maximum.

*Box1. Summary of recommendations for tailoring strength training prescriptions for clients with rheumatoid arthritis*

1. Provide education and resources to support clients’ knowledge of the disease and benefits of strength training.
2. Clear and documented instructions for the exercise prescription should be provided in case the client is experiencing symptoms that cause memory loss or “brain fog.”
3. Screen for co-morbidities and ensure contraindications and cautions to specific co-morbidities are incorporated into the program. For example, avoid the Valsalva maneuver among individuals with high blood pressure.
4. Use adaptive or protective devices such as padded gloves, splints, or adapted shoes to help the client exercise move comfortably, especially with joint deformities.
5. Schedule sessions around peak effectiveness of medication or when symptoms (e.g., pain) are least severe.
6. Strength training can be continued at a lower intensity during a flare, and focus on exercises that involve the least symptomatic joints. Therefore, high intensity exercise testing (e.g., 1 repetition maximum) should be avoided. It may be helpful to have a plan for flare and non-flare days.
7. Ask or assess how the client is feeling (e.g., joint symptoms, pain, stiffness) at the beginning and throughout the session to determine if the program needs to be adapted.
8. Offer variety/adaptability in type of equipment and exercises, range of motion, volume, progression, and frequency in the strength training program.
9. Include a warm-up that increases blood flow to the working muscles and improves safe range of motion. The warm-up can be used to help the client gauge the current disease status that day and to mitigate existing pain and stiffness.
10. Monitor symptoms for a few days after the session to inform whether intensity needs to be decreased in the next session.
11. Pain location (i.e., in the muscle) may be used to help distinguish normal delayed onset muscle soreness pain from potentially harmful joint pain.
12. To help maintain the program, progress slowly to help overcome fear of being active, incorporate activities that the client enjoys, are convenient, and provide variety. Suggest exercising with a friend or joining a group class, and use behavior change techniques such as goal setting and action planning to help your client stick to their goals.
13. If the client’s needs for strength training exceed the clinician’s scope and experience, the client should be referred to a more appropriate exercise professional.

### Quality Appraisal

#### Guidelines

The highest average AGREE II domain score was given for clarity of presentation (95%) the lowest average domain score (19%) was given for applicability. The domain scores of each guideline are reported in Table 4. The mean overall quality rating across the 10 included guidelines was 4.3 (SD 0.9) out of a maximum score of 7.

**Table 4.** Mean AGREE II Domain Scores for Guidelines^a^

ID (Ref)	Domain 1 Score: Scope and Purpose (%)	Domain 2 Score: Stakeholder Involvement (%)	Domain 3 Score: Rigor of Development (%)	Domain 4 Score: Clarity of Presentation (%)	Domain 5 Score: Applicability (%)	Domain 6 Score: Editorial Independence (%)	O1 Rate the overall quality of this guideline	O2 I would recommend this guideline for use
^37^	28	0	2	94	17	0	3	Yes
^38^	28	6	2	100	17	0	3	Yes
^39^	100	56	19	78	17	100	4	Yes
^13^	100	83	77	100	17	75	5	Yes
^58^	100	50	21	94	13	100	4	Yes
^20^	100	94	85	100	46	25	6	Yes
^35^	56	33	29	94	13	75	4	Yes
^45^	83	78	79	89	0	50	5	Yes
^36^	100	61	44	100	50	0	5	Yes
^51^	100	56	65	100	0	25	4	Yes
Average	80	52	42	95	19	45	4.3	

^a^Guidelines included (a) a systematic literature review including a subsequent synthesis of the evidence and a structured consensus process completed by a representative committee (S3), (b) a systematic literature review and synthesis of the evidence only (S2e), (c) a structured consensus process completed by a representative committee only (S2k), or (d) an informal consensus process by a group of experts (S1).

#### Systematic Reviews

Three of the five appraised review articles did not explicitly state that they adhered to a pre-established protocol (Question 2). All five reported some features of a comprehensive literature search (Question 4). Only one provided a list of excluded studies and justified the exclusions (Question 7). All but one assessed the risk of bias (RoB) in their included studies (Question 9). All three meta-analyses used appropriate methods for statistical combination of results (Question 11). Four of the five appraised articles provided some account for RoB when framing and discussing the results of the review (Question 13). Finally, two of the three meta-analyses investigated publication bias and discussed its impact on the results of the review (Question 15). Critical domain scores for the 5 appraised review articles are shown in Table 5.

**Table 5.** AMSTAR-2 Assessment of Systematic Reviews According to Critical Domains.

ID (Ref)	Question 2: Protocol registration before commencement of the review	Question 4: Adequacy of the literature search	Question 7: Justification for excluding individual studies	Question 9: Risk of bias from individual studies included in the review	Question 11^a^: Appropriateness of meta-analytical methods	Question 13: Consideration of risk of bias when interpreting the results of the review	Question 15^a^: Assessment of presence and likely impact of publication bias
^46^	Yes	Yes	No	Yes	Yes	Yes	Yes
^7^	No	Yes	No	Yes	Yes	Partial yes	Yes
^54^	Yes	Yes	Yes	Yes	Yes	Yes	No
^19^	No	Partial yes	No	Yes	N/A	Partial yes	N/A
^50^	No	Partial yes	No	No	N/A	No	N/A

^a^Question 11 and Question 15 only applied to systematic reviews with quantitative analysis.

## Discussion

### Recommendations for Tailoring Strength Training Prescriptions for People with RA

This review highlights available recommendations for tailoring strength training prescriptions for people with RA. Findings span recommendations that address an individuals’ capability, opportunity, and motivation to participate in strength training.^56^ While the scope of recommendations to address participation factors (e.g., barriers and facilitators) was diverse, no single article addresses the full range of factors that affect strength training participation among people with RA. This is supported by the finding that “applicability” of the reviewed literature, which considers the extent that articles provide tools for application and discuss practical considerations such as barriers to implementation, was the lowest-scored AGREE II domain.^33^ Furthermore, many participation factors (e.g., sleep, fatigue, medication side effects, mental health status) lack evidence for recommendations that adequately target these in the context of strength training.

### Clarifying Pain Recommendations

Recommendations regarding pain monitoring were at times conflicting. Pain duration was recommended to be monitored with both 2 hours^42,43^ and 24 hours suggested as a threshold for when to be concerned about pain.^13,40^ Of note, the 24-hour threshold is supported by a guideline^13^ that scored high on the AGREE-II rigor of development domain, while other sources were non-systematic reviews. However, neither recommendation was grounded in empirical evidence. Not only is the range of recommendations for acceptable pain duration post strength training confusing and untested, these recommendations conflict with evidence on delayed onset muscle soreness (DOMS). DOMS is a type of pain that appears 12 to 48 hours after exercise but is considered to be normal, self-limited, and not necessarily a reflection of undesirable damage.^57–60^ This means that timelines for potentially harmful pain and normal DOMS-related pain overlap, potentially creating confusion if pain duration recommendations are not paired with further indicators to distinguish acceptable vs harmful pain. For example, to distinguish harmful pain from typical post strength training pain, recommendations included the use of pain location to distinguish joint pain from expected strength training muscle soreness such as DOMS.^39,44^ Therefore, clarity is needed to contextualize pain monitoring recommendations to prevent confusion or fear amongst people with RA participating in strength training.

### Using Assessment to Guide Safe and Appropriate Prescription

Many of the included recommendations suggested using symptoms to guide strength training prescription, but specifics on how to use findings from symptom assessment to inform prescriptions were lacking. For example, it was recommended to assess joint symptoms, pain, and stiffness to determine whether the program needs adaptation on a given day.^37,39,43^ Using the warm-up was also recommended to assess how the patient is feeling.^39^ Disease activity and symptom fluctuation is a common experience amongst individuals with RA^61^ and a challenge for prescribing strength training, although little evidence is available to guide practice.^30^ Assessment of other factors beyond disease activity and symptoms may also be important for supporting clients in strength training participation. Recommendations from the European League Against Rheumatism (EULAR) advise comprehensive assessments of physical, social, and psychological factors to adapt programs.^13^ Likewise, evidence on which factors to assess and how to assess them in the context of strength training has not been described to date. This practice is still in its infancy and further research is needed on what assessments can be used to individualize and adapt strength training prescriptions. In the interim, this process will require ongoing trial and error and collaboration between professionals and their patients.

### Patient-Level Factors Remaining to be Addressed

Strength training research and recommendations would benefit from exploring how factors such as sleep, mental health status, fatigue, and medication side effects can be managed to facilitate participation in strength training. The broader literature acknowledges these RA-specific participation factors and their interconnected relationship.^36,62–67^ Fatigue has been shown to be associated with mental well-being and sleep disturbance in patients with RA.^68^ Also, medications such as methotrexate and prednisone can be associated with side effects including fatigue and sleep disturbance, as well as weight gain, respectively.^64,69^ Many of the included articles in this scoping review noted strength training’s positive effects on mental health and emotional status.^39,50,51^ However, previous work on general physical activity has acknowledged the potential role of addressing mental health early in the program so that patients with RA can experience these mental health benefits.^18^ Overall, these participation factors have complex relationships amongst themselves and with strength training. Future research should explore how to optimally prescribe strength training in the face of these complex participation factors while considering the unique interconnected relationships at play.

### Strengths/Limitations

A few limitations must be acknowledged. First, the inclusion criteria of guidelines, recommendations, and reviews precludes the ability to examine the evidence of relationships between the prescription recommendations and strength training participation. In other words, it is unclear whether applying these recommendations for strength training prescription leads to increases in strength training participation. This may be an area for future research. Second, the broad nature of the research question required including articles of variable quality. This resulted in some conflicting statements in Table 2 which summarizes prescription recommendations made in each article. To address this issue, the summary of recommendations has been contextualized within the quality of their sources in the discussion where appropriate and the use of the AGREE II or other guidelines are encouraged to promote higher methodological quality in future research or guidelines. Third, only articles with full text written in English were included meaning articles in other languages may have been excluded. A strength of this scoping review was the use of an integrated knowledge translation approach, engaging both patient and healthcare professionals throughout the review process, helping improve the relevance to patients with rheumatologic conditions and their healthcare professionals.

## Conclusion

This review summarizes recommendations for practitioners to tailor RA-specific strength training prescriptions. The physical, psychological, and social factors that affect strength training participation in individuals with RA makes prescribing strength training a complex and multi-faceted endeavor. Indeed, providing information on strength training prescription parameters needs to be paired with how to deliver strength training in the face of these multi-faceted participation factors. Addressing the identified areas for future research may better equip healthcare professionals to usefully prescribe and support patients with RA to experience the many benefits of strength training.

## Supplemental Material

Supplemental Material - Tailoring Strength Training Prescriptions for People with Rheumatoid Arthritis: A Scoping Review Supplemental Material for Tailoring Strength Training Prescriptions for People with Rheumatoid Arthritis: A Scoping Review by Michael L. Wu, B.Kin, Jasmin K. Ma, PhD, B.Kin, Karen Tsui, BSc(PT), Alison M. Hoens, MSc, BSc(PT), and Linda C. Li, PhD, BSc(PT) in American Journal of Lifestyle Medicine

Supplemental Material - Tailoring Strength Training Prescriptions for People with Rheumatoid Arthritis: A Scoping Review Supplemental Material for Tailoring Strength Training Prescriptions for People with Rheumatoid Arthritis: A Scoping Review by Michael L. Wu, B.Kin, Jasmin K. Ma, PhD, B.Kin, Karen Tsui, BSc(PT), Alison M. Hoens, MSc, BSc(PT), and Linda C. Li, PhD, BSc(PT) in American Journal of Lifestyle Medicine

## Note

1
https://www.arthritisresearch.ca/our-team/arthritis-patient-advisory-board/
