# Supplementary material for: Tailoring Strength Training Prescriptions for People with Rheumatoid Arthritis: A Scoping Review
Source: Am J Lifestyle Med. 2022 Sep 9;18(2):200–15. doi: 10.1177/15598276221125415 (PMC10914594; doi:10.1177/15598276221125415)
Supplement: Supplemental Material - Tailoring Strength Training Prescriptions for People with Rheumatoid Arthritis: A Scoping Review [file sj-pdf-1-ajl-10.1177_15598276221125415.pdf]

Tailoring strength training prescriptions for people with rheumatoid arthritis: A scoping review

*Michael L. Wu;[1] *Ma, J.K.;[1,2] Tsui, K.;[1] Hoens, A.M.;[1,2] Linda C. Li;[1,2]

[1]*University of British Columbia, 2329 West Mall, Vancouver, BC, Canada*
[2]*Arthritis Research Canada, 230-2238 Yukon Street, BC, Canada*

Journal: American Journal of Lifestyle Medicine

Correspondence: Jasmin Ma
Arthritis Research Canada
Jasmin.ma@ubc.ca

Table of Contents

1. Sample database search in Medline Ovid

Sample database search Medline Ovid

1. resistance training.mp. or exp Resistance Training/
2. strength training.mp.
3. weight lifting.mp. or exp Weight Lifting/
4. resistance exercise.mp.
5. strength exercise.mp.
6. 1 or 2 or 3 or 4 or 5
7. rheumatoid arthritis.mp. or exp Arthritis, Rheumatoid/
8. clinical practice guideline*.mp.
9. exp Practice Guideline/ or exp Guideline/ or guideline*.mp.
10. recommendation.mp.
11. CPG.mp.
12. prescription*.mp.
13. exp "Review"/ or review.mp.
14. 8 or 9 or 10 or 11 or 12 or 13
15. 6 and 7 and 14

Tailoring strength training prescriptions for people with rheumatoid arthritis: A scoping review

*Michael L. Wu;[1] *Ma, J.K.;[1,2] Tsui, K.;[1] Hoens, A.M.;[1,2] Linda C. Li;[1,2]

[1]*University of British Columbia, 2329 West Mall, Vancouver, BC, Canada*
[2]*Arthritis Research Canada, 230-2238 Yukon Street, BC, Canada*
Journal: American Journal of Lifestyle Medicine
Correspondence: Jasmin Ma
Arthritis Research Canada
Jasmin.ma@ubc.ca

Strength Training for People with Rheumatoid Arthritis: Barriers and Facilitators

Jasmin K Ma,[1,2] Lamiah Adamjee,[2] Jon A Collins,[1] Eileen Davidson,[1] Kelly English,[1] Alison M Hoens,[1] Karen Tsui,[1] Shanon McQuitty,[1] Louella Sequeira,[1] Linda C Li,[1,2]

[1] Arthritis Research Canada, [2] University of British Columbia

Background

- As little as 1-14% of people with rheumatoid arthritis are participating in strength training (Iversen et al., 2014).

Objective

To examine patient-identified barriers, facilitators, and tailoring considerations for designing interventions to improve strength training participation among people with rheumatoid arthritis

Methods

- **Semi-structured interviews:** Interviews were co-developed with nine patient partners. Questions were iteratively modified to reflect new knowledge gleaned from interviews that were coded at mid-way points in the data collection.
- **Analysis:** Inductive thematic coding was used. Peer checking amongst researchers and patient partners was conducted to ensure credibility. We continued the interviews until content saturation was reached in the analysis.
- Themes were mapped onto the COM-B model which proposes that capability, opportunity, and motivation are the three factors necessary for enacting a behaviour, such as strength training (Michie et al., 2011)

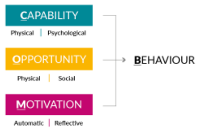

Figure 1: COM-B model (Michie et al., 2011)

Results

- **13 participants**
- **Age:** Range=25-70, Mean=47+/-15
- **Gender:** 3 males, 10 females
- **Geographic location:** 2 rural, 11 urban
- **Strength training experience:** 5=no experience, 5=some experience but less than the guidelines, 3=exceeding the guidelines (2 days/week or more)
- **Perceived RA severity:** 2=severe, 4=moderate, 7=well-controlled

Results (Cont'd)

Automatic Motivation (habits, unconscious)	Reflective Motivation (planned, conscious)	Physical Capability (bodily skills/ability)	Psychological Capability (mental skills/ability)	Physical Opportunity (environment, resources)	Social Opportunity (the people around you)
					Trainer/client relationship
					Practical support from others
			Knowledge of benefits		Group exercise
			ST knowledge	Diverse strengthening activities	The social gym environment
			Understanding the disease	Transportation to a facility	Accountability to others
	Autonomy		Mental health	Time/ priority	Role models
	Behavioural strategies		Disease-responsive planning	Use of technology	Healthcare provider and trainer collaboration
	ST requires focus	Recovery strategies	Memory	Flexible location	Unsupportive ST deliverer
	Taking small steps	Medication effects	Equipment knowledge	The physical gym environment	Healthcare provider recommendations
Intimidation	Reframing/acceptance	Disease management strategies	Identifying good pain vs bad pain	Cost	Networks with shared experiences
Interest	Confidence	Disease symptoms	Disease-responsive exercise prescriptions	RA-specific resources, classes and programs	Others' misunderstanding of the disease
Fear of causing damage	Experiencing benefits of ST	Ability to perform ST technique	Experimenting/ knowing limits to ST	Equipment	ST deliverers trained in RA

Figure 2: Factors that affect strength training participation among people with rheumatoid arthritis. RA=rheumatoid arthritis, ST=strength training.

Conclusion

- There are challenges to strength training that are unique to people with rheumatoid arthritis
- COM-B analysis revealed almost 50 factors that affect strength training participation across capability, opportunity, and motivation

This information has been modified from a poster presented at American College of Rheumatology, Philadelphia, USA, 2019: Ma J, Collins J, Davidson E, et al. Strength training for people with rheumatoid arthritis: Barriers, facilitators, and tailoring considerations. *Arthritis Rheumatol*. 2019;71(Supplement 10):3928-3929
